# Electrical Stimulation of C6 Glia-Precursor Cells In Vitro Differentially Modulates Gene Expression Related to Chronic Pain Pathways

**DOI:** 10.3390/brainsci9110303

**Published:** 2019-10-31

**Authors:** Ricardo Vallejo, David C. Platt, Jonathan A. Rink, Marjorie A. Jones, Courtney A. Kelley, Ashim Gupta, Cynthia L. Cass, Kirk Eichenberg, Alejandro Vallejo, William J. Smith, Ramsin Benyamin, David L. Cedeño

**Affiliations:** 1 Millennium Pain Center, Bloomington, IL 61704, USA; rvallejo@treatingpain.com (R.V.); cklumbrera@gmail.com (C.A.K.); ashim6786@gmail.com (A.G.); ccass95@gmail.com (C.L.C.); avlumbrera@gmail.com (A.V.); William.J.Smith.MED@dartmouth.edu (W.J.S.); rbenyamin@treatingpain.com (R.B.); 2 Department of Psychology, Illinois Wesleyan University, Bloomington, IL 61701, USA; 3 Department of Chemistry, Illinois State University, Normal, IL 61790, USA; dcplatt@ilstu.edu (D.C.P.); majone3@ilstu.edu (M.A.J.); keichen0311@gmail.com (K.E.); 4 Department of Biology, Illinois Wesleyan University, Bloomington, IL 61701, USA; jrink@iwu.edu; 5 South Texas Orthopaedic Research Institute, Laredo, TX 78045, USA; 6 Geisel School of Medicine, Dartmouth College, Hanover, NH 03755, USA; 7 College of Medicine, Department of Surgery, University of Illinois at Urbana-Champaign, Champaign-Urbana, IL 61801, USA

**Keywords:** electrical stimulation, oxidative stress, glial cells, gene expression, cell culture

## Abstract

Glial cells comprise the majority of cells in the central nervous system and exhibit diverse functions including the development of persistent neuropathic pain. While earlier theories have proposed that the applied electric field specifically affects neurons, it has been demonstrated that electrical stimulation (ES) of neural tissue modulates gene expression of the glial cells. This study examines the effect of ES on the expression of eight genes related to oxidative stress and neuroprotection in cultured rodent glioma cells. Concentric bipolar electrodes under seven different ES types were used to stimulate cells for 30 min in the presence and absence of extracellular glutamate. ES consisted of rectangular pulses at 50 Hz in varying proportions of anodic and cathodic phases. Real-time reverse-transcribed quantitative polymerase chain reaction was used to determine gene expression using the ∆∆C_q_ method. The results demonstrate that glutamate has a significant effect on gene expression in both stimulated and non-stimulated groups. Furthermore, stimulation parameters have differential effects on gene expression, both in the presence and absence of glutamate. ES has an effect on glial cell gene expression that is dependent on waveform composition. Optimization of ES therapy for chronic pain applications can be enhanced by this understanding.

## 1. Introduction

The initiation, propagation, and perception of acute pain has been traditionally described in terms of the excitation of neuronal transmission pathways [1]. Although these pathways represent an integral process responsible for the organism’s perception of pain, they are unable to adequately explain the mechanism by which acute pain progresses into chronic pain. It has been demonstrated that, in response to injury, neurons and glial cells sustain nociceptive signals via a variety of neurotransmitters, cytokines, and neuropeptides [2]. A recent explanation of the progression from acute to chronic pain implicates the activation of glial cells adjacent to the site of injury or inflammation in addition to sensitization of neurons [3]. During the process of neural sensitization, which is central to the development of chronic pain, glial cells are activated, and undertake an immunoresponsive role characterized by the release of chemical signals such as cytokines and chemokines that induce neuronal hyperexcitability, inflammation, and apoptosis [4]. This process involves pro-inflammatory processes associated with the development of the chronic pain state, although it is also known that the activated glia can also release anti-inflammatory modulators indicative of a neuroprotective role [5]. 

Previous work has demonstrated that glial cells can respond to electrical stimulation. Roitbak and Fanardjian [6] showed that the membrane of glia can be depolarized in a manner dependent of stimulation parameters such as frequency and intensity. Work by Lee and coworkers [7,8] demonstrated that electrical stimulation of astrocytes can induce the release of the excitatory neurotransmitter glutamate, dependent on the characteristics of the stimulating waveform. Furthermore, electrical stimulation of glial cells can modulate their interaction with their surrounding neurons or other glial cells. For example, Lee et al. [9] showed that glutamate released by astrocytes is responsible for suppression of the spindle activity of thalamic neurons. Yamazaki et al. [10] showed that the depolarization of oligodendrocytes induced by electrical stimulation increases the conduction of action potential of cortical pyramidal neurons myelinated by the stimulated oligodendrocyte. 

Electrical stimulation of neural tissue has been proven effective for relief of chronic neuropathic pain. The mechanism of action is centered on the effect of the electrical field on action potentials transmitted by neurons [11]. Our group has proposed that the mechanism of action must account for the modulation of glial cells, considering the crucial role of these cells in the development and maintenance of chronic neuropathic pain and their responsiveness to electrical stimulation [12]. Furthermore, glia are the largest cell population of cells in neural tissues clinically accessed for epidural stimulation of the dorsal columns of the spinal cord (SCS) in the lower thoracic segments (T8–T11). Our group recently determined that glial cells outnumbered neurons (20:1) in these spinal segments [13]. Animal models of neuropathic pain have also shown that SCS modulates the expression of genes involved in various biological processes relevant to pain. The results from these studies suggest that the potential mechanism of action of SCS is mediated by modulation of the expression of specific genes [14,15,16].

In this study, rodent C6 glioma cells were used to investigate the effects of different waveforms including monophasic, and charge-unbalanced and charge-balanced biphasic waveforms on expression of genes related to oxidative stress and cell protection in glial cells. C6 glioma cells possess progenitor properties that can express oligodendrocytic and astrocytic phenotypes, and have been widely used as a model system for studying conditions and factors that play a vital role in proliferation and differentiation of glial cells [17,18,19]. Extracellular glutamate was added to culture medium to induce stress in the C6 cells in order to simulate an in vitro injury model [20]. Glutamate is an excitatory neurotransmitter which acts as a neuronal-glial signal activating glial cells via one of three types of glutamate receptors [21]. Glutamate has been used extensively to induce stress and cytotoxicity in C6 glioma cells to understand its mechanism of action and to study the protective nature of several agents to ameliorate glutamate-induced toxicity [22,23,24]. 

The inclusion of novel waveforms with varying parameters will help us determine the importance of each feature in modulating gene expression. Here we evaluated the effect of varying electrical stimulation (ES) waveforms on expression of eight genes related to different biological processes including immune function: *Gfap*; synaptic transmission: *Slc7a11*, *Glul*; neuroprotection: *S100a4*; oxidative stress processes: *Mt2a*, *Gsr*, *Hmox1*; and cell adaptive responses to stressful stimulation: *Bag3*.

## 2. Materials and Methods

Monosodium glutamate was obtained from Sigma Aldrich (St. Louis, MO, USA) and used as provided, at a concentration of 10 mM in order to induce stress in the cultured glioma cells. A 100 mM stock solution was prepared by dissolving in deionized water. This solution was filter sterilized with a MILLEX^®^ GP 0.22 μm syringe driven filter unit (Millipore, Cork, Ireland) before using in the cell cultures. 

**Cell Culture:** Axenic *Rattus norvegicus* C6 glioma cells (ATCC CCL-107, Manassas, VA, USA) were grown in sterile 6-well plates using high glucose Dulbecco’s Modified Eagle’s Medium (incomplete DMEM) (Sigma Life Sciences D6429; St. Louis, MO, USA) supplemented with 15% (*v*/*v*) horse serum (ATCC; Manassas, VA, USA), and 5% (*v*/*v*) heat treated fetal bovine serum (GIBCO; Waltham, MA, USA) designated as “complete medium” [25]. Cells were grown at 37 °C under a 5% CO_2_ atmosphere in the presence of an open vessel of water to maintain relative humidity. To transfer the adherent cells, trypsin (Sigma Life Sciences T4049; St. Louis, MO, USA) was used to release cells from the bottom of the well in accordance with the manufacturer’s instructions. Trypsin was subsequently neutralized upon addition of “complete medium” by the α-1 antitrypsin present from the serum supplementation of the incomplete DMEM. This cell preparation was then centrifuged (Labnet Hermle Z 400K; Edison, NJ, USA) at 2000 rpm for 10 min at 7 °C. The supernatant was discarded, and the resultant cell pellet was re-suspended in “complete medium” and plated as required. Large cultures of cells were maintained in CELLSTAR^®^ TC sterile 6-well plates (Greiner Bio-one, Monroe, NC, USA). For experimentation, cells were harvested, as previously described, re-suspended in “complete medium”, and applied to sterile 96-well cell culture plates (Falcon^®^; Corning, NY, USA) in a total volume of 100 µL per well. Experiments began when the cells in the 96-well plates were deemed confluent. All cell culturing was performed in a UV sterilized hood (Thermo Electron Corporation Forma Class II Biological Safety Cabinet; Waltham, MA, USA) for maintaining sterile conditions.

**Electrical Stimulation:** Four concentric bipolar electrodes (FHC Microelectrodes; Bowdoin, ME, USA) were arranged in parallel and connected to either a current isolator (WPI A365; Sarasota, FL, USA) slaved to an arbitrary waveform generator (Siglent SDG1025; Shenzen, China), or a current-controlled external neurostimulator (Medtronic Intellis 97725; Minneapolis, MN, USA). Electrical stimulation (ES) consisted of rectangular pulses delivered at a frequency of 50 Hz with an intensity of 0.15 mA for 30 min using one of seven different waveforms (see Table 1). Two waveforms were charge-unbalanced monophasic pulses with either a cathodic or anodic pulse width of 50 µs. Two waveforms were charge-unbalanced asymmetric biphasic waveforms with a cathodic pulse width of 50 µs and anodic pulse width of either 100 µs (AsymBi 1-2) or 25 μs (AsymBi 1-0.5). Three waveforms were charge-balanced. One was an actively balanced biphasic symmetric (SymBi 1:1) waveform with 50 µs pulse width in each phase, and two were passively balanced with either a cathodic front (Cathodic PR) or an anodic front (Anodic PR) with a pulse width of 60 µs in the leading phase. 

C6 glioma cells (ATCC) were grown in 96-well plates with 100 µL “complete medium” per well. A custom-built stimulation apparatus created using the 123D Design CAD software and printed on a MakerBot Replicator+ 3D printer housed the concentric bipolar electrodes and situated them in an appropriate orientation such that the electrode tip was submerged in the medium, yet elevated above the bottom of the plate (see Figures S1 and S2 in the Supplementary Material). The files for this apparatus are freely available on thingiverse.com (thing: 3614719). 

Two hours prior to stimulation, the medium was changed to incomplete DMEM, with or without added glutamate to a 10 mM concentration. A post-stimulation incubation was carried out for two hours at 37 °C in 5% CO_2_ atmosphere to allow for the uptake of the additional glutamate by the cells. For each experiment, two 96-well plates were seeded using a single pool of glioma cells in “complete medium”. One plate contained cells cultured with no glutamate addition, while the other plate, contained cells cultured with an additional 10 mM glutamate. Additionally, each 96-well plate had four wells of cells designated to account for cell viability using the MTT assay. Cells were added to plates a minimum of 12 h before the experiments were conducted to allow cells to adhere and spread to indicate normal glial cell morphology. ES was applied at room temperature in ambient atmosphere for 30 min with the four replicate wells to be pooled treated simultaneously.

In order to obtain sufficient RNA in a single independent sample for quantitative determination of gene expression, four replicate wells for each treatment condition were pooled before undergoing RNA extraction. RNA quantification was obtained from three (*n* = 3) independent experiments, each consisting of one pool of four wells that were treated simultaneously under similar experimental conditions.

**MTT Cell Viability Assay:** To establish whether aliquots of a single cell pool, delivered via an 8-channel pipettman, yielded sub-cultures in a reproducible fashion, an MTT cell viability assay was used. The 3-(4,5-dimethylthiazol-2-yl)-2,5-diphenyltetrazolium bromide (MTT; Sigma Aldrich, St. Louis, MO, USA) assay was utilized, as described in the literature [25], with slight modifications to assess glioma cell viability. A confluent population of glioma cells, from a 6-well plate, was trypsinized and then suspended in “complete medium”, creating a single pool of cells. Aliquots ranging from 0–100 µL were seeded into a 96-well plate. “Complete medium” was then added to the wells such that each well had a final volume of 100 µL of medium. Plates were incubated for 24 h prior to performing the MTT cell viability assay at which point the medium was replaced with incomplete DMEM, diluted 1:10 with filtered sterilized saline. MTT incubation occurred at room temperature for a duration of 45 min. Absorbance (A) was measured at 595 nm using a microplate reader (Bio-Rad iMark, Hercules, CA, USA). Determination of viability was conducted in quadruplicate. The effect of ES on cell viability was obtained relative to the viability of cells not exposed to ES (viability = A_ES_/A_No-ES_) and are reported as a mean ± 95% Confidence Interval. Absorbance values were corrected by subtracting the absorbance value of medium-only (negative control) wells. 

**Light Microscopy:** To establish that the parameters of the electrical field applied during these experiments were not causing substantial cell death, light microscopy was used to visualize the cell population before and after ES. Visual assessment of the morphology and distribution of the glioma cells was performed using an inverted light microscope (Jenco USA; Portland, OR, USA) under 40× magnification. Images were captured with a digital camera (Asus ZOOM3, AsusTek Computer Inc.; Taipei, Taiwan).

**Real-Time Quantitative Reverse-Transcriptase Polymerase Chain Reaction (RT-qPCR):** After the two-hour post-stimulation incubation, the incomplete DMEM was removed and the glioma cell RNA from four pooled wells was extracted using Tri-Reagent (Sigma Aldrich; St. Louis, MO, USA) following the manufacturer’s instructions. Isolated RNA was treated with DNase I (1 U/10 µL reaction volume, Thermo Fisher Scientific, Waltham, MA, USA) in the presence of RNase inhibitor (Thermo Fisher Scientific, Waltham, MA, USA) at 1 U/µL in a 30 µL reaction volume. RNA was column-purified (Thermo Fisher Scientific, Waltham, MA, USA) following manufacturer’s instructions, and quantitated using a spectrophotometer (DS-11, DeNovix, Wilmington, DE, USA). Isolated RNA samples were stored in 75% ethanol at −80 °C until further use. RNA (300 ng) was reverse-transcribed into first strand cDNA using a High-Capacity cDNA Reverse Transcription Kit as per manufacturer’s instructions (Applied Biosystems, Foster City, CA, USA). First strand cDNA was diluted to 160 ng/µL and stored at −20 °C until use. 

Gene expression was quantitated in real time in triplicate with a thermal cycler using the manufacturer’s instructions (AriaMx, Agilent Technologies, Santa Clara, CA, USA), using SYBR Green dye with low Rox (Agilent Technologies, Santa Clara, CA, USA). Gene-specific primers used are shown in Table 2.

Gene-of-interest transcripts were identified in the UCSC genome database for *R. norvegicus* using the July 2014 update [28]. Primers were designed using optimized settings in NCBI’s Primer-BLAST tool [29]. Primer characteristics were verified using OligoCalc [30] and checked for off-target amplification using the ThermoFisher Scientific Multiple Primer Analyzer tool (ThermoFisher Scientific; Waltham, MA, USA). The gene-specific transcript profile for each sample was determined by analyzing C_q_ values via the ΔΔC_q_ method [31]. Briefly, C_q_ values were normalized to *Gapdh* to obtain a ΔC_q_ value. The ΔC_q_ values for each set of trials were averaged and normalized to the corresponding control group in order to obtain the ΔΔC_q_. To evaluate the effect of glutamate, the control groups were either No-ES No-Glu (for No-ES groups) or ES No-Glu (for ES groups). For the effect of stimulation, the control groups were either No-ES No-Glu (for No-Glu groups) or No-ES Glu (for Glu groups). Fold changes were obtained from the corresponding ΔΔC_q_ values. 

**Statistical Analysis:** For RT-qPCR, data are reported as the average fold change (average of high and low fold change) determined by adjusting ΔΔC_q_ values with their corresponding standard error of mean (ΔΔC_q_ ± SEM). Statistical analyses were performed using SigmaPlot 12.5 (Systat Software, San Jose, CA, USA). Normality of the data was assessed using the Shapiro–Wilk method. Comparisons were based on ΔC_q_ values obtained for a given gene due to ES relative to the corresponding control values and carried out using one-way analysis of variance followed by post-hoc corrections for multiple comparisons (Holm–Sidak method). A value of *p* < 0.05 was considered significant.

## 3. Results

### 3.1. MTT Cell Viability Assay 

**Figure 1:**
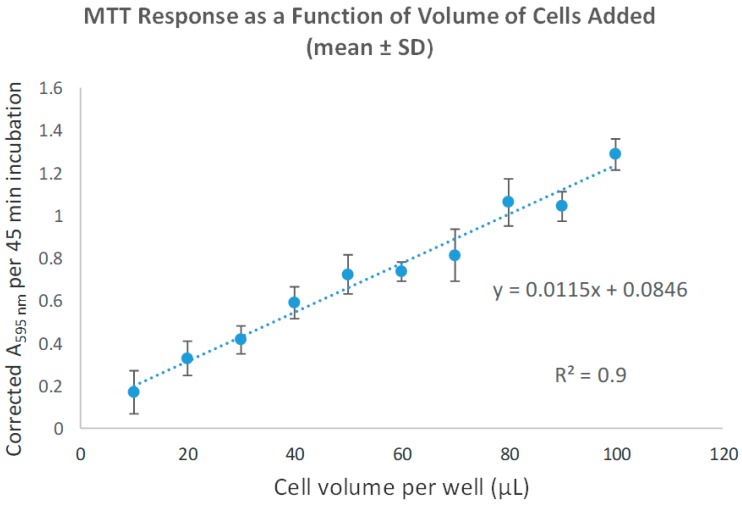
Results (mean ± SD; *n* = 4) from the serial dilution of a glial cell pool representative experiment. Data are shown by plotting the corrected A_595nm_ per 45 min value as a function of volume of cell pool added to the well (with the volume of complete DMEM also added to yield a final volume of 100 µL; for example, if 20 µL of cell pool was seeded then 80 µL of medium was also added).

The corrected average of four replicates taken at 595 nm absorbance is plotted in Figure 1. This shows that glioma cells were delivered with reproducibility (*R*^2^ = 0.976) and the consistency of the MTT value from experiment to experiment is a first approximation of use of same cell number and/or cell viability. When comparing the ES to no ES groups, MTT values indicated an average of 97.0% relative viability. ES groups produced cell viabilities in the 85%–118% range: Cathodic PR (118% ± 8%), AsymBi 1:2 (113% ± 24%), SymBi 1:1 (112% ± 8%), Monophasic Anodic (105% ± 6%), Monophasic Cathodic (94% ± 2%), Anodic PR (90% ± 13%), and AsymBi 1:0.5 (85% ± 4%). 

### 3.2. Light Microscopy

**Figure 2:**
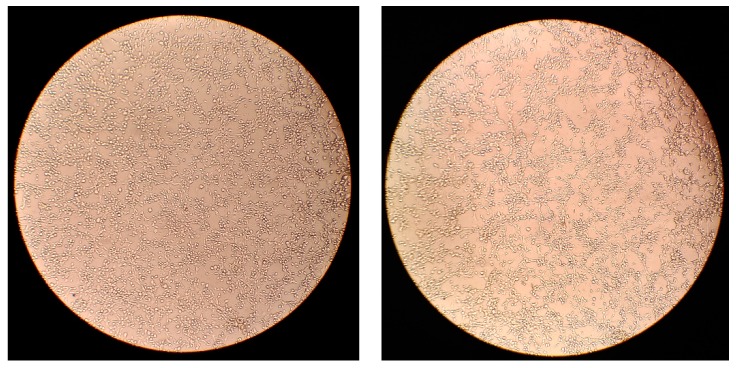
Light microscopy images (40×) of the same cell population before Cathodic PR ES (**left**) and after ES (**right**).

As shown in Figure 2, visualization of a representative cell population revealed little to no change in cell morphology or distribution of cells on a plate after electrical stimulation was applied, indicating the stimulation parameters were reasonably safe for cell vitality. 

### 3.3. RT-qPCR

The effect of the addition of 10 mM glutamate on gene expression without electrical stimulation was evaluated. The expression of 6 out of 8 genes was significantly modulated by incubating the cell with additional glutamate (Table 3). Three of them (*Slc7a11*, *Mt2a* and *Hmox1*) were upregulated and three downregulated (*Bag3*, *S100a4*, and *Gsr*). Table 4 shows that ES significantly modulates the expression of genes in cell cultures that had been stressed by the addition of glutamate. The modulation was waveform dependent. 

The effect of ES alone was also determined without additional glutamate (Table 5). Similarly, the effects were waveform dependent. In the absence of glutamate, 5 out of 8 genes (all but *Gsr*, *Hmox1*, and *Bag3*) were significantly or near significantly modulated by ES, while in the presence of 10 mM Glu, 6 out of 8 genes (all but *S100a4* and *Bag3*) were significantly modulated. Additionally, the effect of ES with glutamate on gene expression was evaluated relative to that in cells that were not exposed to ES and additional glutamate. This allows for evaluating the combined effects of experimental variables. As expected, gene expression was significantly modulated in a waveform dependent manner (Table 6).

## 4. Discussion

**Effect of Glutamate:** Three genes were significantly upregulated by the addition of 10 mM glutamate to the cell cultures: *Slc7a11* (2.9-fold), *Mt2a* (1.8-fold), and *Hmox1* (2.0-fold), while three genes were significantly downregulated: *S100a4* (0.65-fold), *Gsr* (0.64-fold), and *Bag3* (0.80-fold). Neither *Gfap* nor *Glul* were significantly modulated. Excess extracellular glutamate is transported into the cells and causes intracellular oxidative stress due to the perturbation of the redox balance of the cells [22]. The increase in the expression of *Hmox1* (codes for heme oxygenase-1), a well-known indicator of oxidative stress in cells [32], and *Mt2a* (codes for metallothionein 2a), a well-established natural antioxidant [33], seems to result as a response to incubating cells with 10 mM glutamate. This is corroborated by the increase in the expression of *Slc7a11*, the gene that codes to the glutamate/cystine antiporter gene, which is involved in the maintenance of intracellular glutathione balance and has been associated with extracellular glutamate regulation [34]. Since SLC7A11 favors secretion of glutamate in exchange of cystine, an excess of extracellular glutamate induces an increase in the expression of this transporter in order to remove any of such glutamate that is transported into the cells in exchange for cystine (cysteine dimer). The balance of intracellular cysteine and glutamate is crucial to regulate the levels of glutathione and glutathione-disulfide, a key system for cellular redox balance. Overexpression of *Slc7a11* has been associated with antioxidant activity. The excess glutamate also induces the decrease in the expression of *Gsr* that codes for glutathione-disulfide reductase, an enzyme that catalyzes the formation of glutathione from its oxidized species. The decrease of *Bag3* by glutamate is also an indication of cell stress, as a reduction of the protein encoded by this gene (BAG3) is associated with caspase-mediated apoptotic processes [35]. Finally, *S100a4* has been shown to have a neuroprotective role [36]. Thus, a decrease in its expression implies a disruption of its protective function induced by glutamate.

**Effect of ES in the Absence of Additional Glutamate:** When evaluating the effect of ES on cell cultures without additional glutamate, it is evident that the response is dependent on the waveform. Anodic pulses that are passively balanced (Anodic PR) affected the expression of most genes by upregulating *Slc7a11* (2.0-fold) and *Mt2a* (2.9-fold). In contrast, cathodic pulses that are passively balanced (Cathodic PR) tend to upregulate *Mt2a* (2.5-fold, *p* = 0.092). Interestingly, using cathodic pulses that are actively balanced (SymBi 1:1) did not affect the expression of any of the genes significantly. Among the charge-unbalanced waveforms, anodic monophasic pulses downregulated *S100a4* (0.46-fold) significantly, while *Glul* (0.59-fold, *p* = 0.069) was also downregulated almost significantly. On the other hand, cathodic monophasic pulses tended to downregulation of *S100a4* (0.63-fold, *p* = 0.063) and *Mt2a* (0.044-fold). The asymmetric cathodic pulses with larger anodic content (AsymBi 1:2) also downregulated *S100a4* (0.62-fold), as well as *Gfap* (0.49-fold), while the asymmetric cathodic pulses with lower anodic content (AsymBi 1:0.5) did not affect gene expression significantly. These results imply that the method utilized for charge balancing as well as the choice of a cathodic or an anodic leading phase influences the outcome. For instance, an anodic waveform with passive balancing affects more of the genes studied than a balanced cathodic equivalent and a cathodic waveform with active balancing. Indeed, in general, the expression of the majority of the genes studied trend similarly when utilizing passive balance and differently from active balance. Recently, we reported that the anodic content in a waveform affected the in vivo expression of genes, relevant to pain processes mediated by glial cells, in spinal cord tissue that had been electrically stimulated within a rodent model of neuropathic pain [37]. The current study did not yield strong correlations between anodic content and fold changes in expression when using monophasic cathodic (0% anodic), AsymBi 1:0.5 (33%), SymBi 1:1 (50%), AsymBi 1:2 (67%) and monophasic anodic (100%). There are, however, some interesting trends. For example, the expression of *S100a4*, *Glul*, and *Mt2a* tend to decrease with an increase in anodic content, when the effect of monophasic cathodic pulses (no anodic content) is excluded. Monophasic ES (cathodic and anodic) tend to induce similar effects on gene expression. Interestingly, in the case of *Hmox1*, monophasic ES and anodic ES with passive recharge tend to increase the expression, while the other waveforms tend to decrease it. These results indicate that the choice of anodic vs. cathodic stimulation, the anodic content of a cathodic waveform, and how charge balance is done can influence the expression of certain genes associated with redox processes in a particular manner.

**Effect of ES in Cell Cultures Previously Incubated with Additional Glutamate:** The previous sections imply that both glutamate and ES affect the expression of genes and that for ES this is dependent on the characteristics of the waveform. This is also observed in cell cultures that were incubated with additional glutamate and then exposed to ES relative to those that were incubated with glutamate but not exposed to ES (Table 4). Among the charge-balanced waveforms, the Anodic PR waveform produced significant upregulation of *Gsr* (2.2-fold), and *Glul* (1.9-fold), while all the other genes tend to be upregulated as well (notably, *Slc7a11* 1.9-fold, *p* = 0.083). The cathodic PR waveform significantly upregulated *Gfap* (4.3-fold), *Slc7a11* (2.5-fold), and *Mt2a* (2.6-fold). In contrast, the actively balanced SymBi 1:1 waveform did not produce significant modulation. This is consistent with the results discussed in the previous section that indicates that the mode of charge balancing has a distinctive effect on gene expression. Among the unbalanced waveforms, the monophasic cathodic one upregulated *Slc7a11* (2.3-fold), while the monophasic anodic one did not affect the genes significantly. In general, monophasic pulses tend to upregulate the expression of the studied genes. Interestingly, an unbalanced cathodic waveform with more cathodic content (AsymBi 1:0.5) upregulated *Gfap* (2.6-fold, *p* = 0.050) and downregulated *Hmox1* significantly (0.40-fold). The unbalanced cathodic waveform with larger anodic content (AsymBi 1:2) seems to have a counter effect in the expression of these two genes, as well as *Mt2a*, which was downregulated (0.47-fold, *p* = 0.058), and tend to be upregulated by the other asymmetric waveform. This result suggests a correlation between anodic content in waveforms and some gene expressions. Correlations including all waveforms, except passively balanced ones, were not very strong, although they provide some interesting trends. For instance, the expression of *Gfap*, *Slc7a11*, *Glul*, and *Mt2a* are inversely correlated with anodic content (0% for monophasic cathodic to 100% for monophasic anodic). The expression of *S100a4*, *Hmox1*, and *Bag3* are positively correlated, while there is no correlation for *Gsr*. 

**Figure 3:**
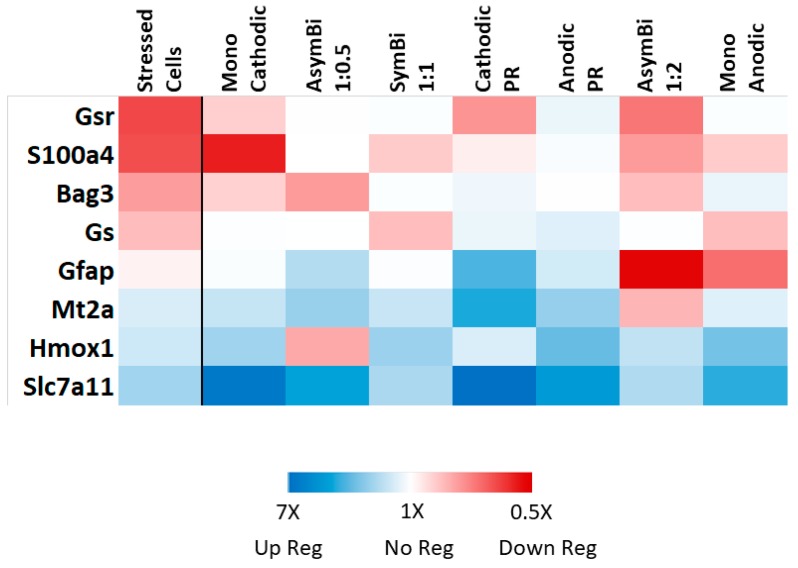
Heat map illustrating the effect of gene expression by glutamate alone (Stressed Cells column) and ES on stressed cells relative to the expression of untreated control cells (No-Glu_No-ES). White color indicates gene expression equivalent of untreated control cells.

It is evident from Table 6, in which gene expression in cells stressed with glutamate that have been exposed to ES (ES_Glu) is compared with control cells (No-ES_No-Glu) that there is a general trend toward a reversal of gene expression due to ES. This is illustrated in the heat map shown in Figure 3, where the effect of ES is contrasted with the observed effect of glutamate to cells in the absence of ES (i.e., No-ES_Glu vs. No-ES_No-Glu, Table 3). In general, ES upregulated gene expression in cells that had been stressed with glutamate (Table 4). Therefore, most ES waveforms further up regulated *Mt2a*, *Hmox1*, and *Slc7a11*, which had been significantly upregulated by glutamate. Given the antioxidant role of these three genes, the results imply that ES enhances the antioxidant activity of the cells. Furthermore, the genes that had been significantly downregulated by glutamate (*Gsr*, *S100a4*, and *Bag3*), which can negatively affect neuroprotection, were modulated to levels similar to the control cells (No-ES_No-Glu).

## 5. Conclusions

Glial cells, a primary component of the central nervous system, have a diverse set of functions including their involvement in the development and maintenance of chronic pain [12,38]. From a mechanistic standpoint, the effectiveness of ES therapy is lacking elucidation. To explore the mechanism(s) of action of the electrical stimulation treatments, experiments in this study were designed to test electrically stimulated glioma cells in culture for changes in gene expression. The results indicated that glutamate induced changes in gene expression consistent with its known effect as a promoter of cell stress including oxidative stress. ES waveforms had differential effects on gene expression that are favorable in terms of reducing the effects of glutamate. In general, ES enhances the antioxidant response of the cells and modulates the expression of other genes back towards the expression of unstressed cells. Furthermore, properties of ES waveforms, such as the polarity of the leading pulse, the anodic content, and the way charge balance is obtained may be used to regulate their modulatory effect on genes and their antioxidant and cell protective properties.

## Figures and Tables

**Table 1:** Electrical stimulation parameters used in this study.

Group Name	Stimulation Parameters	Charge Balance	Duty Cycle	Cathodic Charge	Anodic Charge	Waveform Shape
Monophasic Cathodic	F = 50 Hz PW = 50 μs	Unbalanced	0.25%	7.5 nC	0	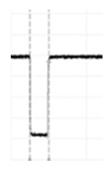
Asymmetric Biphasic 1:0.5 (AsymBi 1:0.5)	F = 50 Hz PW = 50 μs cathodic, 25 μs anodic	Active unbalanced	0.38%	7.5 nC	3.8 nC	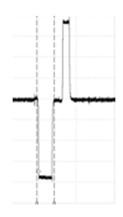
Symmetric Biphasic 1:1 (SymBi 1:1)	F = 50 Hz PW = 50 μs per phase	Active balanced	0.50%	7.5 nC	7.5 nC	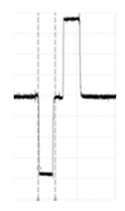
Cathodic PR	F = 50 Hz PW = 60μs ^‡^	Passive balanced	5% *	9.0 nC	9.0 nC	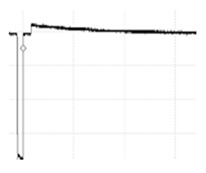
Anodic PR	F = 50 Hz PW = 60 μs ^‡^	Passive balanced	5% *	9.0 nC	9.0 nC	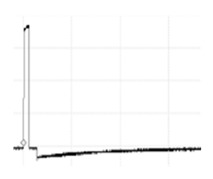
Asymmetric Biphasic 1:2 (AsymBi 1:2)	F = 50 Hz PW = 50 μs cathodic, 100 μs anodic	Active unbalanced	0.75%	7.5 nC	15 nC	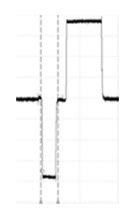
Monophasic Anodic	F = 50 Hz PW = 50 μs	Unbalanced	0.25%	0	7.5 nC	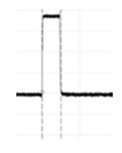

F = Frequency, PW = pulse width. Charges are mean values. Duty Cycle = (PW_cathodic_ + PW_anodic_) × F. *, PW for balancing phase approximated to be around 1 ms. ^‡^, PW of leading phase.

**Table 2:** Primer sequences and amplification metrics for RT-qPCR analyses. Expected Amplicon Length is the expected size in base pairs (bp) of the PCR product. If not otherwise specified, primers were designed and validated in-house. Primer sequences previously reported: *Gapdh* in Vallejo et al. [26]; and *Mt2a* in Sabolic et al. [27].

Gene	Codes for	Accession Number	Sequence	Direction	Role
*Gfap*	Glial fibrillary acidic protein	NM_017009	5′-CAGGAAATTGCTGGAGGGCGAA-3′	Forward	Immune function
			5′-CTTGAGGTGGCCTTCTGACACAG-3′	Reverse	
*Slc7a11*	Glutamate cystine transporter	NM_001107673	5′-ACAAACGCCCAGATATGCATCGTC-3′	Forward	Synaptic transmission
			5′-GGTGCTAAACGGATCCGAGTAAAGG-3′	Reverse	
*Glul*	Glutamine synthetase	M29579	5’-GCCTTCTAATGGCTTCCCTGGAC-3’	Forward	Synaptic transmission
			5’-ACCTCGGCATTTGTCCCTGTG-3’	Reverse	
*S100a4*	S100 calcium binding protein A4	NM_012618	5′-GCACTTCCTCTCTCTTGGTCT-3′	Forward	Neuro-protection
			5′-GTCTGTCCTTCTCCCCAGGA-3′	Reverse	
*Mt2a*	Metallothionein 2A	NM_001137564	5′-CACAGATGGATCCTGCTCCT-3′	Forward	Redox process
			5′-AAGTGTGGAGAACCGGTCAG-3′	Reverse	
*Gsr*	Glutathione-disulfide reductase	NM_053906	5′-GATGTATCACGCTGTGACCACGAG-3′	Forward	Redox process
			5′-AGCATCTCATCGCAGCCAATCC-3′	Reverse	
*Hmox1*	Heme oxygenase 1	NM_012580	5′-TGCTCGCATGAACACTCTGGAG-3′	Forward	Redox process
			5′-GACTCTGGTCTTTGTGTTCCTCTGTC-3′	Reverse	
*Bag3*	Bcl2 associated athanogene 3	NM_001011936	5′-CAGACAGATAAACAGTGTGGACAGGTG-3′	Forward	Cell adaptive response
			5′-AGGACGAGGATGAGCAGTCAGAG-3′	Reverse	
*Gapdh*	Glyceraldehyde-3-phosphate dehydrogenase	NM_017008	5′-CTCATGACCACAGTCCATGC-3′	Forward	House-keeping/Control
			5′-TTCAGCTCTGGGATGACCTT-3′	Reverse	

**Table 3:** RT qPCR gene expression represented as average fold change evaluating the effect of the addition of glutamate in cell cultures without stimulation (No-ES_Glu).

Biological Process	Gene	No-ES_Glu vs. No-ES_No-Glu
Immune Function	*Gfap*	0.965
Synaptic Transmission	*Slc7a11*	2.859 *
	*Glul*	0.847
Neuroprotection	*S100a4*	0.653 *
Redox Processes	*Mt2a*	1.797 *
	*Gsr*	0.639 *
	*Hmox1*	1.991 *
Cell Adaptive Response	*Bag3*	0.801 *

*p* < 0.05 was considered significant. *, represents significance vs. no-glutamate no-stimulation (No-ES_No-Glu).

**Table 4:** RT qPCR gene expression represented as average fold change evaluating the effect of stimulation in cell cultures with the addition of glutamate (ES_Glu).

Biological Process	Gene	ES_Glu vs. No-ES_Glu						
		Monophasic Cathodic	AsymBi 1:0.5	SymBi 1:1	Cathodic PR	Anodic PR	AsymBi 1:2	Monophasic Anodic
Immune Function	*Gfap*	1.125	2.531 ^‡^	1.099	4.335 *	1.931	0.532	0.723
Synaptic Transmission	*Slc7a11*	2.274 *	1.108	0.928	2.477 *	1.903 ^‡^	0.907	1.589
	*Glul*	1.198	1.171	1.001	1.596	1.889 *	1.221	0.998
Neuroprotection	*S100a4*	0.861	1.492	1.351	1.432	1.710	1.221	1.351
Redox Processes	*Mt2a*	1.185	1.588	1.166	2.574 *	1.660	0.470 ^‡^	0.914
	*Gsr*	1.386	1.592	1.655	1.263	2.154 *	1.133	1.667
	*Hmox1*	1.421	0.401 *	1.468	0.879	1.910	1.128	1.795
Cell Adaptive Response	*Bag3*	1.120	0.975	1.319	1.654	1.231	1.076	1.794

Anodic content in the waveform increases from left to right. *p* < 0.05 was considered significant. * represents significance vs. no-stimulation with glutamate (No-ES_Glu); ^‡^ denotes 0.05 ≤ *p* < 0.10.

**Table 5:** RT qPCR gene expression represented as average fold change evaluating the effect of stimulation in cell cultures without glutamate (ES_No-Glu).

Biological Process	Gene	ES_No-Glu vs. No-ES_No-Glu						
		Monophasic Cathodic	AsymBi 1:0.5	SymBi 1:1	Cathodic PR	Anodic PR	AsymBi 1:2	Monophasic Anodic
Immune Function	*Gfap*	0.821	0.745	0.802	1.469	1.653	0.488 *	0.700
Synaptic Transmission	*Slc7a11*	0.914	0.690	0.596	1.204	1.924 ^‡^	0.843	0.921
	*Glul*	0.659	1.469	1.068	1.111	1.108	0.724	0.580 ^‡^
Neuroprotection	*S100a4*	0.632 ^‡^	1.072	0.923	1.162	1.468	0.617 ^‡^	0.449 *
Redox Processes	*Mt2a*	0.521	1.310	1.392	2.445 ^‡^	2.875 *	0.722	0.444
	*Gsr*	0.875	0.654	1.134	1.179	1.106	0.807	0.980
	*Hmox1*	1.342	0.679	0.625	0.712	1.136	0.701	2.029
Cell Adaptive Response	*Bag3*	0.948	0.939	0.880	1.102	1.484	1.154	1.057

Anodic content in the waveform increases from left to right. *p* < 0.05 was considered significant. *, represents significance vs no-stimulation no-glutamate (No-ES_No-Glu); ^‡^ denotes 0.05 ≤ *p* < 0.10.

**Table 6:** RT qPCR gene expression represented as average fold change evaluating effect of stimulation with glutamate (ES_Glu) relative to cells not exposed to ES and additional glutamate (No-ES_No-Glu).

Biological Process	Gene	ES_Glu vs. No-ES_No-Glu						
		Monophasic Cathodic	AsymBi 1:0.5	SymBi 1:1	Cathodic PR	Anodic PR	AsymBi 1:2	Monophasic Anodic
Immune Function	*Gfap*	1.085	2.442 *	1.061	4.184 *	1.864 ^‡^	0.513 ^‡^	0.698
Synaptic Transmission	*Slc7a11*	6.502 *	3.170 *	2.653 *	7.083 *	5.443 *	2.592 *	4.545 *
	*Glul*	1.015	0.991	0.848	1.352	1.600	1.034	0.846
Neuroprotection	*S100a4*	0.563	0.975	0.883	0.935	1.118	0.798	0.883
Redox Processes	*Mt2a*	2.129	2.855 *	2.095	4.627 *	2.983 *	0.845	1.643
	*Gsr*	0.886	1.017	1.058	0.807	1.377	0.724	1.065
	*Hmox1*	2.830 *	0.798	2.923 *	1.750	3.804 *	2.246 *	3.574 *
Cell Adaptive Response	*Bag3*	0.897	0.781	1.057	1.325	0.986	0.862	1.437

Anodic content in the waveform increases from left to right. *p* < 0.05 was considered significant. * represents significance vs. no-stimulation no-glutamate (No-ES No-Glu) ^‡^ denotes 0.05 ≤ *p* < 0.075.

## Supplementary Materials

The following are available online at https://www.mdpi.com/2076-3425/9/11/303/s1.
