# Supplementary material for: Electrical Stimulation of C6 Glia-Precursor Cells In Vitro Differentially Modulates Gene Expression Related to Chronic Pain Pathways
Source: Brain Sci. 2019 Oct 31;9(11):303. doi: 10.3390/brainsci9110303 (PMC6896182; doi:10.3390/brainsci9110303)
Supplement: Supplementary file 1 [file brainsci-09-00303-s001.pdf]

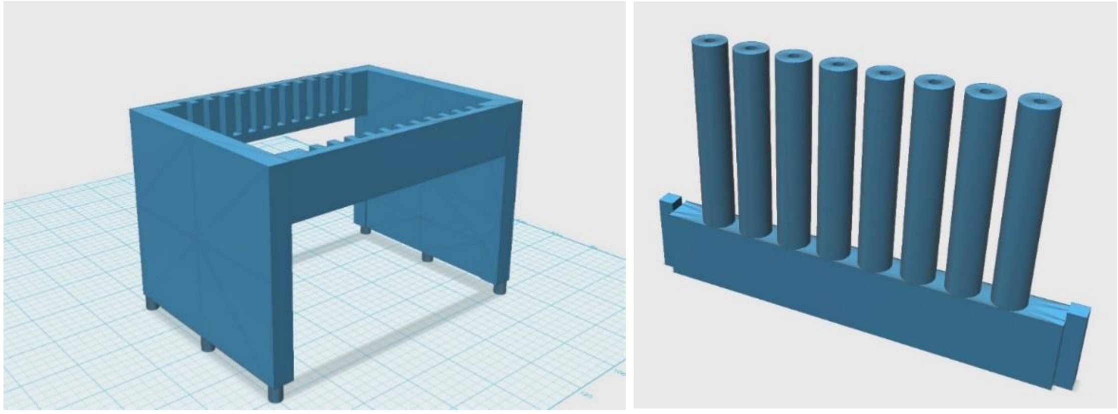

Figure S1. Digital representation of the electrode holder (right) and stimulation apparatus (left). The grooves in the apparatus guide the holder into an ideal position for stimulating any given well on a 96-well plate. Both guide and holder were created using the 123D Design CAD software and printed on a MakerBot Repilcator+ 3D printer. The files for this apparatus are freely available on thingiverse.com (thing: 3614719).

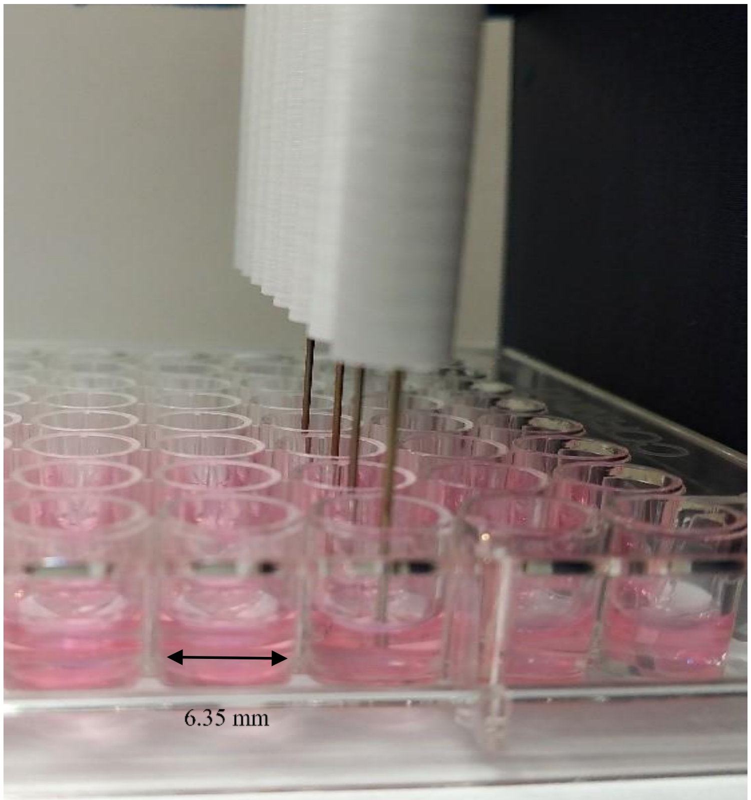

Figure S2. A photopraph showing the electrical stimulation setup. Concentric bipolar electrodes housed within the electrode guide stimulating row 3 of a 96-well plate. The four electrodes are pictured with submerged tips that are suspended in 100 μL volume. Well diameter is 6.35 mm as indicated.
